# Risk-Stratified Radiotherapy in Pediatric Cancer

**DOI:** 10.3390/cancers16203530

**Published:** 2024-10-18

**Authors:** Rituraj Upadhyay, Arnold C. Paulino

**Affiliations:** 1 Department of Radiation Oncology, The Ohio State University Wexner Medical Center, Columbus, OH 43212, USA; rituraj.upadhyay@osumc.edu; 2 Department of Radiation Oncology, University of Texas MD Anderson Cancer Center, Houston, TX 77030, USA

**Keywords:** pediatric cancer, risk-stratified therapy, proton therapy, medulloblastoma, rhabdomyosarcoma

## Abstract

While the cure rate of cancer in children has markedly improved in the last few decades, late effects continue to be a problem in survivors. Radiotherapy, which is a major component of treatment in many cancers, is one of the major agents responsible for late toxicity. In the past decade, radiotherapy has been omitted in patients achieving excellent response to chemotherapy, such as in Hodgkin lymphoma and some Wilms tumors with lung metastases. Likewise, response to chemotherapy has been used to determine whether lower doses of radiation can be delivered in intracranial germinoma and pediatric nasopharyngeal carcinoma. Molecular subtyping in medulloblastoma is currently being employed, and in WNT-pathway M0 tumors, the reduction in radiotherapy dose to the craniospinal axis and tumor bed is currently being investigated. Finally, dose escalation was recently evaluated in patients with rhabdomyosarcoma > 5 cm who do not achieve a complete response to initial 9 weeks of chemotherapy as well as for unresectable Ewing sarcoma patients to improve local control.

## Simple Summary

We discuss the role of risk-stratification and personalized treatment for various pediatric cancer patients, with the goal being to improve tumor control and decrease late effects of radiation in long-term survivors. We discuss settings in which radiation can be safely omitted or de-escalated, such as Hodgkin lymphoma, Wilms tumor with lung metastases and WNT pathway Medulloblastoma, and settings that warrant treatment escalation such as larger tumors with rhabdomyosarcoma or Ewing sarcoma, poor responders to chemotherapy and oligometastatic disease settings. We also summarize currently enrolling COG and other cooperative group trials.

## 1. Introduction

Radiation therapy (RT) is one of the primary treatment modalities for pediatric cancer treatment. Surgery and/or chemotherapy may also play a role depending on histology, patient age, and tumor location. The emergence of modern systemic therapy regimens has resulted in de-escalation in the dose and volume of radiation for several pediatric cancers. In addition, modern RT techniques such as proton therapy and stereotactic ablative body radiotherapy (SABR) offer decrease in treatment-related late effects, and improve the quality of life for long-term survivors of pediatric cancers [1,2,3]. In addition, over the last one to two decades, there have been rapid advancements in precision medicine with the genomic and molecular characterization of pediatric cancers [4,5]. This has led to further biology-driven risk-stratification of tumors such as medulloblastoma and ependymoma, and has helped in personalizing treatment options. Despite developments in targeted therapies, RT remains the mainstay in the management of many pediatric cancers in the primary as well as adjuvant settings, with new data also suggesting a role in the management of metastatic cancers. Risk-stratified radiotherapy involves tailoring treatment intensity based on individual risk factors, including the type of cancer, stage, genetic profile, and patient-specific factors such as age and comorbidities, with the goal of administering the minimum effective dose of radiation while preserving or improving outcomes. We hereby discuss the role of modern risk stratification in tailoring the treatment paradigm for pediatric cancers, specifically the tailoring of RT based on prognostic factors and treatment response. Given that pediatric tumors encompass a wide spectrum of histologies, we discuss the most frequently encountered tumors that commonly require radiotherapy as part of definitive management.

## 2. Assessment of Prognostic Factors

**Figure 1:**
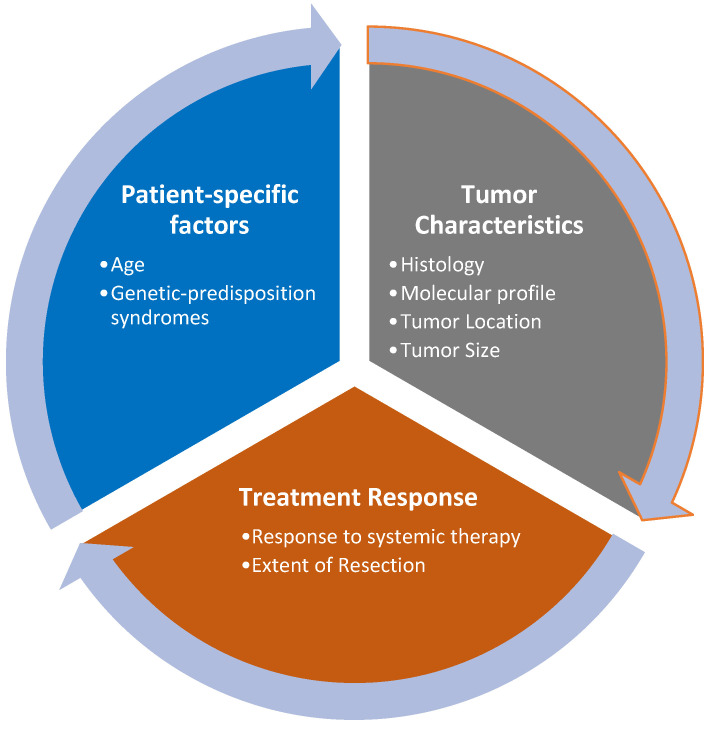
Factors affecting risk stratification.

Risk stratification for personalized radiation treatment is based on prognostic factors that can be tumor-specific or patient-specific, as described in Figure 1.

### 2.1. Tumor Characteristics

Several tumor-specific factors can influence outcomes, such as the histology and molecular profile. Certain tumor types and their genetic mutations may influence radiation sensitivity and resistance. Molecular profiling helps in identifying tumors that may respond better or worse to radiation. In addition, tumor location and size can often aid in determining adequate radiation doses, such as for rhabdomyosarcomas. Tumors in proximity to critical structures may require more precise targeting with intensity modulated proton therapy (IMPT) to reduce damage to surrounding healthy organs.

### 2.2. Patient-Specific Factors

The age of the patient is often essential in determining radiation dose. Younger children are at higher risk for developmental and growth-related side effects. For these reasons, patients with medulloblastoma younger than 3 years are typically not treated with craniospinal irradiation (CSI), and CSI is delayed until they are 3 years or older. In addition, genetic predisposition syndromes can influence the radiation sensitivity. For example, some genetic syndromes such as Li-Fraumeni syndrome can increase susceptibility to radiation-induced secondary cancers.

Tumor response to treatment is one of the most important prognostic factors which may help escalate or de-escalate treatment. Table 1 and the next sections detail the current ongoing and active Children’s Oncology Group (COG) studies evaluating risk-stratified RT in common pediatric malignancies.

## 3. De-Escalation of Radiotherapy Using Molecular Characterization

### 3.1. Medulloblastoma

Medulloblastoma is the most common embryonal CNS tumor in the pediatric population [6]. Patients usually present with a posterior fossa mass with obstructive hydrocephalus. Management involves a multimodal approach including maximal safe resection followed by CSI, tumor bed boost and multiagent systemic therapy [7,8]. Current adjuvant treatment guidelines revolve around risk grouping, which is based on patient age, amount of post-operative residual disease, metastatic disease, and tumor histology. OS at 5 years for patients with standard-risk disease (residual disease ≤1.5 cm^2^, ≥3 years old, and no metastatic disease) is ~80%, and it is ~60% for those with high-risk disease (residual disease >1.5 cm^2^, metastatic disease) [9]. The majority of patients who are long-term survivors experience late toxicities, which can include neuroendocrine dysfunction, growth defects, cognitive effects, ototoxicity and secondary cancers [10,11,12]. Strategies to decrease late effects revolve around treatment de-escalation, especially by reducing the CSI radiation dose.

Traditional risk-adapted CSI for patients with standard-risk and high-risk disease involves 23.4 Gy and 36–39.6 Gy, respectively [13]. This is followed by a boost to the tumor bed to a total dose of 54 Gy. In addition, any metastatic sites can receive further doses depending on size and location. Early efforts to omit or reduce the dose of CSI resulted in worse patient outcomes [14,15]. For younger children <3 years, RT is typically delayed until the age of 3 years or older, given the incidence of significant neurocognitive deficits in this patient population [16,17]. The recently published ACNS 0331 study evaluated the deintensification of CSI dose and volume with standard-risk medulloblastoma. They found that reducing the radiation boost volume to involved field (IFRT) instead of entire posterior fossa was safe and did not compromise survival. But a reduction in CSI dose from 23.4 Gy to 18 Gy in young children (3–7 years), although associated with better neurocognitive outcomes, resulted in inferior survival outcomes [18]. For younger children <2 years of age, the COG trial ACNS0334 evaluated high-dose chemotherapy regimens and peripheral blood stem cell rescue with high-risk medulloblastoma or CNS embryonal tumors, and the results suggest that the omission of CSI upfront does not appear to compromise survival, although focal radiotherapy may be reasonable for select patients (NCT00336024) [19].

Molecular subtyping in medulloblastoma is currently being employed for risk stratification, and has revolutionized the management paradigm of these tumors [20]. Current stratification shows that the disease falls into one of four groups: wingless (WNT), sonic hedgehog (SHH), Group 3, and Group 4 [5,21]. These groupings have prognostic implications, with WNT patients having the best prognosis and Group 3 having the worst, with current standard treatment guidelines. Estimated 5-year progression-free survival (PFS) for the WNT subgroup is >90%, followed by SHH (80%), Group 4 (70%) and Group 3 (50–60%) [22,23]. In addition, within these subgroups, patients with MYC or MYCN amplification have worse outcomes and are typically considered high-risk [24].

Current studies are focused on molecular risk-directed therapy. Treatment de-intensification is being considered for lower-risk subgroups, such as with further reduction of RT dose to the craniospinal axis and tumor bed for WNT-pathway M0 tumors. The recently closed COG study ACNS1422 (NCT02724579) evaluated whether CSI dose can be reduced to 18 Gy and chemotherapy intensity can be reduced in patients with average risk WNT-pathway tumors who have positive Beta-catenin and the presence of CTNNB1 [exon 3] mutations, without large-cell/anaplastic medulloblastoma or MYC/MYCN amplification. The FOR-WNT2 trial is also evaluating low-dose CSI (NCT04474964). The SJMB12 evaluated a reduced CSI dose of 15 Gy in this same population (NCT01878617). They presented their initial data (abstract only) of patients with M0 WNT tumors treated with 15 Gy CSI and 51 Gy primary site boost, and reported a high 5-year PFS and OS of 90.4% and 98.6%, respectively, with fewer treatment-related side-effects [25]. However, a pilot study omitting CSI entirely for WNT-driven medulloblastoma has closed due to inferior outcomes with all patients relapsing in <1 year and needing salvage CSI (NCT02212574) [26]. In Europe, the possibility to deliver a reduced CSI dose of 18 Gy to a select subgroup of children with a low-risk biological profile is being investigated in the ongoing International Society of Pediatric Oncology (SIOP) PNET-5 study (NCT02066220). In addition, trials such as SJMB12 also investigated intensified treatment regimens for patients in higher-risk subgroups, including the addition of gemcitabine and pemetrexed for those with high-risk Group 3 or Group 4 medulloblastoma, and targeted SHH inhibitor therapy for those with SHH-positive medulloblastoma [27]. The awaited results of these ongoing studies will aid in molecularly informed patient selection for decreasing late effects.

### 3.2. Ependymomas

Ependymomas are common pediatric brain tumors usually diagnosed in young children at a median age of four years. The mainstay of treatment in children with localized disease is maximal safe resection followed by RT (54–59.4 Gy) to the tumor bed [28]. Postoperative RT significantly improves event-free survival (EFS) to about 77% at 7 years after a sub-total resection and 88% after gross total resection (GTR) [29,30]. Given favorable outcomes in some ependymomas after GTR, there may be benefits to avoiding radiation in select patients with low risk ependymomas. Multiple trials have evaluated the role of adjuvant chemotherapy to avoid or delay RT and to be used as a salvage therapy, with variable outcomes [31,32]. The COG ACNS0121 study evaluated observation of classic supratentorial ependymoma after GTR (Stratum 1, 3%); chemotherapy, second surgery and RT for those undergoing subtotal resection (Stratum 2, 18%); and immediate postoperative radiation for anaplastic supratentorial or infratentorial tumors after near-total resection (Stratum 3, 33%) or GTR (Stratum 4, 46%). Of 11 patients who were observed after GTR, 5 patients failed locally, with a 5-year EFS of 61.4% and 5-year OS of 100% [33]. In patients with anaplastic histology who received immediate postoperative RT after GTR (*n* = 141, 40%), the 5-year EFS was 60.7%, which was improved compared to historical outcomes after delayed RT [33].

Recent understanding of the molecular subtypes of ependymomas has provided more insight into the biological behavior of these tumors [21]. Among supratentorial ependymomas, the ZFTA/RELA fusion subtype tumors are considered more aggressive than YAP fusion or subependymoma [34]. For posterior fossa tumors, group A (PFA) subtype and 1q gain are poor prognostic factors, while PFB tumors are much less likely to recur or metastasize [34,35]. Recent evidence suggests adjuvant radiation may not add significant benefit for patients with PFB ependymomas after GTR, who have a 5-year PFS of 91% compared to 81% for PFA ependymomas [36]. In the ACNS0121 study, the 5-year EFS was 65–70% after GTR compared to 37% for incompletely resected tumors with adjuvant chemotherapy and delayed RT (Stratum 2). Although the extent of resection remains one of the most important prognostic factors, current studies are evaluating molecular risk stratification in combination with histology and extent of resection to re-evaluate patients where deferring or omitting radiation can be considered to avoid additional late toxicities. The role of adjuvant chemotherapy was investigated in ACNS0831, the most recent ependymoma protocol from COG (NCT01096368). This study evaluated post-operative focal RT alone or RT + four cycles of maintenance chemotherapy in children with ependymoma after GTR or near total resection. Final results have not been published yet, but preliminary results suggest similar 3-year EFS with (*n* = 164) or without maintenance chemotherapy (*n* = 161) in patients with gross total and near-totally resected ependymoma treated with post-operative focal RT on intention-to-treat analysis (78% vs. 72%, *p* = 0.074), but improved 3-year EFS on “as treated” analysis (80% vs. 71%, *p* = 0.012) [37]. In this study as well, patients with grade 2 supratentorial ependymoma after GTR were assigned to observation (*n* = 37), and had a 5-year EFS of 66.9% and 5-year OS of 100%.

## 4. De-Escalation of Radiotherapy after Excellent Response to Chemotherapy

### 4.1. Hodgkin Lymphoma (HL)

The management of HL has evolved over the last several decades to optimize cure and minimize the risk of treatment-related late effects. The radiation treatment volumes have evolved from extended-field RT in the early 1970s (5y OS 80%) to involved site-radiation therapy in modern times, with 5y OS of >95% with a decreased risk of long-term effects [38,39]. Given favorable outcomes in patients with early-stage disease, the concept of risk-adapted therapy is well elucidated in the treatment of HL, with a goal of avoiding unnecessary therapy for individuals with a lower risk of disease progression or recurrence, while providing more intense upfront therapy for individuals with high-risk disease [40,41].

Contemporary treatment paradigm for HL is based upon two COG protocols—AHOD0431 for low-risk HL (stage IA or IIA non-bulky disease) and AHOD0031 for intermediate-risk (stage IA or IIA with bulky disease; or stages IB, IAE, IIB, IIAE, IIIA, IVA with or without bulk disease) [42,43]. The COG AHOD0431 study evaluated a response-directed treatment in patients <21 years with low-risk (stage IA or IIA non-bulky) HL [43]. Patients received minimal chemotherapy, with radiotherapy reserved for patients who did not achieve a complete response (CR). At 4 years, 49.0% patients received minimal chemotherapy and no radiation, and the OS rate was 99.6%. Negative positron emission tomography scan after 1 cycle of chemotherapy (PET1) was associated with a favorable EFS outcome. Similarly, the COG study AHOD0031 evaluated the role of early chemotherapy response in tailoring subsequent therapy in pediatric intermediate-risk Hodgkin lymphoma [42]. In this study, patients received two cycles of doxorubicin, bleomycin, vincristine, etoposide, cyclophosphamide, and prednisone (ABVE-PC), followed by response evaluation. Rapid early responders (RERs, patients with ≥60% reduction in size after 2 cycles) received two additional ABVE-PC cycles, followed by CR evaluation (≥80% reduction in size). RERs with CR were randomly assigned to involved-field radiotherapy (IFRT) or no additional therapy; RERs with less than CR received IFRT. Slow early responders (SERs, no RER) were randomly assigned to receive two additional ABVE-PC cycles with or without dexamethasone, etoposide, cisplatin, and cytarabine (DECA). All SERs received IFRT. The 4-year EFS was 85.0%; 86.9% for RERs and 77.4% for SERs (*p* < 0.001). The 4-year OS was 97.8%; 98.5% for RERs and 95.3% for SERs (*p* < 0.001). The 4-year EFS was 87.9% vs. 84.3% (*p* = 0.11) for RERs with CR who were randomly assigned to IFRT vs. no IFRT, and 86.7% versus 87.3% (*p* = 0.87) for RERs with positron emission tomography (PET)-negative results at response assessment. The 4-year EFS was 79.3% vs. 75.2% (*p* = 0.11) for SERs who were randomly assigned to DECA versus no DECA, and 70.7% vs. 54.6% (*p* = 0.05) for SERs with PET-positive results at response assessment. This trial demonstrated that early response assessment supported therapeutic titration (omitting radiotherapy in RERs with CR; augmenting chemotherapy in SERs with PET-positive disease).

Although there has been a nationwide decrease in the use of combined modality treatment for HL, this perhaps reflects the bias of ongoing clinical trials designed to avoid consolidation radiotherapy [44]. In a recent analysis of the National Cancer Database including pediatric patients <21 years with stage I or II HL, patients receiving combined modality treatment had an improved 5-year OS after treatment (97.3% vs. 94.5%, adjusted HR = 0.57; 95% CI, 0.42–0.78; *p* < 0.001) [45]. Oeffinger et al., in a recent study, evaluated the impact of risk-adapted therapy for pediatric HL on long-term morbidity using the Childhood Cancer Survivor Study (CCSS) population [46], and reported that from the 1970s to the 1990s, there was a 20% reduction in the decade-specific risk of a grade-3–5 late effect (HR, 0.8; 95% CI, 0.7 to 0.9, *p* = 0.002). The risk of grade-3–5 events was substantially elevated in survivors who had a recurrence and/or stem cell transplant, similar to that of survivors treated with high-dose, extended-field radiotherapy (HR, 1.2; 95% CI, 0.9 to 1.5). A contemporary regimen for low–intermediate-risk HL was estimated to lead to a 40% reduction in the risk of grade-3–5 events (HR, 0.6; 95% CI, 0.4 to 0.8) compared with survivors treated with chest radiotherapy ≥ 35Gy in combination with an anthracycline or alkylating agent [46]. To summarize, risk-adapted therapy for pediatric HL can help reduce serious long-term adverse events, although the impact on local control remains unclear.

### 4.2. Pediatric Nasopharyngeal Carcinoma

Nasopharyngeal carcinoma (NPC) is an uncommon pediatric malignancy and accounts for only about 2% of all NPCs [47,48]. NPCs in children are typically seen in adolescent males, are associated with Epstein–Barr virus, and present at more advanced stages than adults [47]. The standard of care management in adults includes concurrent chemoradiation with or without induction chemotherapy, with long-term survival of 60–80% [49,50,51]. Recent randomized clinical trials in the adult population have investigated the impact of induction chemotherapy, and the Meta-Analysis of Chemotherapy in Nasopharyngeal Collaborative Group (MAC-NPC) showed the superiority of induction chemotherapy and chemoradiation over chemoradiation alone for PFS, LC, and DC [52]. Although children and adolescents with NPC have been typically excluded from these trials, smaller cooperative group experiences have evaluated the use of induction chemotherapy [53].

The COG study ARAR0331 evaluated response-adapted chemoradiation following induction chemotherapy in children and adolescents with NPC [54]. In this study, patients with Stage IIb-IV NPC received three cycles of cisplatin and fluorouracil, followed by chemoradiation. Patients with complete or partial response to induction received 61.2 Gy to the nasopharynx and neck disease, while patients with stable disease received 70.2 Gy. The 5-year EFS and overall survival (OS) estimates were 84.3% and 89.2%, respectively, and 5-year cumulative incidence estimates of local, distant, and combined relapse were 3.7%, 8.7%, and 1.8%, respectively. About 18.6% patients received 70.2 Gy, 4.1% received doses between 63.9 Gy and 66.6 Gy, and 77.3% received a dose of 61.2 Gy or lower. Although this was a single-arm study, estimates suggests that a radiation dose reduction to 61.2 Gy is possible for patients responding to induction chemotherapy [54]. In addition, despite the more advanced presentation in children, their outcomes seem to be superior to adults [47].

Two more German prospective trials—NPC91 and NPC2003—have studied the de-escalation of radiation dose after induction chemotherapy, boosted by the addition of Interferon-beta after radiation for 6 months [50,55]. The cumulative radiation dose in the NPC2003 study was 54 Gy in patients who achieved complete remission to neoadjuvant chemotherapy and 59.4 Gy in other patients. They reported an EFS of 92.4% and OS of 97.1% at 30 months [55]. In a recent updated analysis, the EFS and OS were 93.6% and 96.7% after a median follow-up of 73 months [56]. To summarize, induction chemotherapy followed by response-adapted chemoradiation is the standard of care in the management of pediatric NPCs with high OS. Radiation doses may be reduced in patients with complete remission after induction chemotherapy, and may limit radiation-related late effects.

### 4.3. Intracranial Germ Cell Tumors (GCT)

Germ cell tumors of the central nervous system are a heterogenous group of tumors most commonly located in the pineal or suprasellar region, presenting at a median age of about 10–12 years [57]. Patients with non-germinomatous GCTs historically have had worse outcomes than those with germinomas, with a 5-year OS of 20–45% with full-dose CSI alone or chemotherapy alone [58,59,60]. As discussed above, CSI in children may be associated with deleterious late effects, such as increased risk of secondary malignancy, endocrinopathies, reduced spinal growth, ototoxicity, and neuropsychological dysfunction [1,10]. Response to chemotherapy has been used to determine whether lower doses of radiation can be delivered for intracranial GCTs.

Recent COG and SIOP studies combining RT and chemotherapy have demonstrated improved OS 75–84%, and support the possibility of de-escalating therapy in a subgroup of patients, especially in a setting of effective chemotherapy options [61,62]. In the SIOP 96 trial (NCT00293358), patients with non-germinomatous GCTs received four courses of chemotherapy (cisplatin, etoposide, and ifosfamide) followed by involved-field RT to 54 Gy (localized disease) or 30 Gy CSI, with a boost to 54 Gy to the primary tumor and sites of macroscopic metastases (metastatic disease) [62]. The initial COG ACNS0122 trial used six cycles of chemotherapy (carboplatin/etoposide alternating with etoposide/ifosfamide), followed by 36 Gy CSI and 54 Gy to the primary tumor bed [61]. In this study, 3-year EFS rates were 92% and 94.1% for patients with localized disease who achieved a CR or PR to chemotherapy, respectively. Based on these data, the ACNS1123 protocol suggests dose de-escalation in patients with localized non-germinoma GCTs who achieve a CR or PR to chemotherapy (NCT01602666). The recommended RT dose was 30.6 Gy whole-ventricular irradiation (instead of CSI), followed by involved-field focal boost to 54 Gy after a CR or PR to chemotherapy [63]. They showed a 3-year PFS of 88% and OS of 92% with this approach, compared to 92% and 94%, respectively, in ACNS0122 [64]. On this study, there was a concern for a distinct pattern of recurrence, as all patients relapsed in the spine. The currently accruing trial, ACNS2021, is investigating outcomes of whole-ventricle and spinal irradiation followed by a boost for those who respond to chemotherapy to reduce rates of spinal relapse, as well as intensified chemotherapy followed by conventional RT for those who do not respond to induction chemotherapy (NCT04684368).

Germinomas comprise two-thirds of GCTs and have a more favorable prognosis [65,66]. They are risk-stratified based on response to chemotherapy as well. The ACNS1123 study also included a germinoma population (stratum 2). Patients received induction chemotherapy (carboplatin/etoposide × 4); those with a CR to chemotherapy receive a decreased dose of 18 Gy whole-ventricular RT followed by a 12 Gy focal boost to the primary site, while those with a PR or stable disease (≤1.5 cm but >0.5 cm suprasellar or >1 cm pineal) after chemotherapy received 24 Gy whole-ventricular RT followed by a 12 Gy focal boost to the primary site. Here, 74 patients achieved CR and received 18 Gy, with a 3-year PFS of 94.5%, and 16 received 24 Gy with a 3-year PFS of 93.8% [67]. The study suggests high rates of chemotherapy responses, and promises reduced RT doses for reducing long-term morbidities in patients with germinoma.

### 4.4. Low-Grade Glioma

Pediatric gliomas are common CNS tumors and vary in their histology and anatomic distribution. The definitive management of low-grade gliomas (LGG) with gross total resection leads to excellent local control rates of about 80% and 5 y OS of ≥95% [68]. However, LGGs are often unresectable owing to the involvement of critical structures such as the hypothalamus, optic pathway, or brainstem [69]. For these unresectable tumors, RT can be used alone or after chemotherapy as a bridge to RT [70,71]. Because patients often respond to chemotherapy, typically, children under age ten are treated with chemotherapy prior to RT [72,73]. In these children, RT can be deferred or delayed due to concerns regarding the effects on the developing brain [74]. Delaying radiation till progression or functional decline has been reported to improve memory, intelligence quotient (IQ), and cognition [75]. However, in patients with gliomas of the optic pathway, vision can be threatened with any tumor growth given the location of these tumors [76,77]. A recent study evaluated the role of early RT in sporadic optic pathway glioma as a vision-preserving therapy for well-selected older patients [78]. They evaluated 38 patients with a median age of 3 years at diagnosis, of which 11 (29%) received early RT, while 27 (71%) were treated primarily with chemotherapy. Blindness-free survival rates were 81% at 5 years and 60% at 8 years for chemotherapy and 100% at 5 and 8 years for early RT (*p* = 0.017), suggesting that early RT, defined as initial or first-line salvage therapy, is superior for visual preservation in appropriately selected patients.

Another strategy to reduce late effects in children is the reduction in CTV margin to 5 mm [79]. The COG study ACNS0221 reported that a 5 mm margin provides acceptable disease control in pediatric patients with LGG [80]. On this trial, 3–21-year-old children with unresectable progressive, recurrent, or residual LGGs received a radiation dose of 54 Gy in 30 fractions with MRI-based planning and a 5 mm anatomically limited CTV margin (*n* = 85); and had a 5-year EFS of 71% and 5-year OS of 93%. Male patients and those with larger tumor size had worse EFS and OS. In terms of dose, 50 to 54 Gy is an accepted standard, and even doses 45 to 50 Gy (in younger patients) can provide a high rate of local control [78].

### 4.5. Wilms Tumor

The management of Wilms tumor (WT) is based on tumor stage and histology, incorporating multimodality approaches, with OS rates of >90% [81]. Radiation dose and volume is dependent on stage, favorable vs. unfavorable histology, and tumor and surgical factors such as preoperative tumor rupture and diffuse peritoneal seeding needing whole-abdomen RT. In the NWTS5 study, patients with stage II-IV focal or diffuse anaplasia received 10.8 Gy to the abdomen or flank, depending on the extent of disease, with a boost of 10.8 Gy to areas of bulky residual tumor [81]. For stage III diffuse anaplastic WT, a higher dose of flank radiation (19.8 Gy vs. 10.6 Gy) improved 4-year EFS from 58% to 73%, and decreased the local relapse rate from 14.8% (in NWTS5) to 6.4% in the AREN0321 study [82].

Patients with stage IV WT often present with lung metastases. Traditionally, all patients with lung nodules suspected to be metastases receive whole-lung irradiation (WLI) 12 Gy in 1.5 Gy fractions (10.5 Gy for patients <12 months old) [83]. The COG AREN0533 study evaluated risk-stratified therapy for patients with favorable histology WT and isolated lung metastases by adjusting treatment based on lung nodule response to induction and loss of heterozygosity (LOH) at 1p and 16q. Patients with incomplete lung nodule response after 6 weeks of therapy or LOH at 1p and 16q received WLI in addition to increased chemotherapy (Regimen M), while those with complete lung nodule response did not receive WLI. The omission of WLI in patients after CR (*n* = 133) resulted in 4-year EFS values of 79.5%, and an excellent 4-year OS was maintained (96.1%) [84]. For incomplete responders after WLI (*n* = 159), 4-year EFS and OS were 88.5% and 95.4% respectively. Overall, compared to the predecessor NWTS5 study, 4-year EFS (85.4% vs. 72.5%, *p* < 0.001) and OS (95.6% vs 84.0% *p* < 0.001) were improved significantly, suggesting that the omission of WLI in select patients with CR to chemotherapy and no LOH 1p and 16q is feasible [84]. In a combined analysis of AREN 0532 and AREN 0533, augmenting therapy for patients with LOH 1p/16q improved 4-year EFS [85].

## 5. Escalation of Radiotherapy Using Poor Response to Chemotherapy

### 5.1. Rhabdomyosarcoma

Rhabdomyosarcomas (RMS) constitutes about 40% of soft tissue sarcomas in children and adolescents [86]. The standard of care management of rhabdomyosarcoma involves a combined modality approach including multi-agent chemotherapy, surgery (if resectable with minimal morbidity) and RT. Risk stratification guides the adequate radiation dose, including histology, primary site, stage, group, response to chemotherapy, and more recently FOXO1 fusion status [87,88]. Cytogenetic studies identify frequent translocations t(2;13) or variant t(1;13) in patients with alveolar RMS, which generate PAX3-FOXO1 or PAX7-FOXO1 fusion genes. FOXO1 fusion is seen in about 80% of alveolar RMS [89]. The analysis of patients in COG trials has demonstrated the prognostic significance of FOXO1 fusion status, but has also shown conflicting results [90,91]. Hibbitts et al. evaluated data from six COG trials to evaluate risk stratification with the addition of FOXO1 fusion status to traditional clinical features, and found that FOXO1 status (positive vs. negative) was a significant predictor of EFS and OS for patients with both localized (EFS 52% vs. 78%; OS 65% vs. 88%) and metastatic disease (EFS 6% vs. 46%; OS 19% vs. 58%) [88]. Based on this study, FOXO1 status can aid in risk stratification and help select patients who may not need radiation, such as Group I FOXO1-negative fusion status.

The COG study D9803 evaluated radiation dose for intermediate-risk RMS [92]. On this study, the recommended RT dose was 36 Gy for clinical group I node-negative (N0) patients or 41.4 Gy for patients with positive margins (clinical group 2) or nodal involvement (N1). A dose of 50.4 Gy was used for the definitive treatment of clinical group III tumors. In the adjuvant setting, patients with negative margins received 36 Gy, whereas those with microscopic residual or biopsy confirmation of complete response received 41.4 Gy, and those with gross residual disease received 50.4 Gy postoperatively. Although the EFS was ~70% for all patients, tumors ≥5 cm were more likely to fail locally than tumors <5 cm (25% vs. 10%, *p* = 0.0004), warranting novel approaches for these patients. The recently completed COG intermediate-risk RMS study, ARST1431, allocates treatments based on FOXO1 fusion testing (NCT02567435). This study is also evaluating the role of Temsirolimus. Radiation dose escalation is considered in patients at greater risk of local failure by increasing the total dose to 59.4 Gy (from 50.4 Gy) for tumors >5cm at diagnosis, for those who do not achieve a complete response to the initial 9 weeks of chemotherapy (NCT02567435) [93].

### 5.2. Ewing Sarcoma (ES)

The management paradigm for ES is similar to that of RMS, with role of induction chemotherapy followed by definitive local therapy (surgery and/or radiation) and maintenance chemotherapy [94]. Patients with unresectable disease are candidates for definitive RT. Standard doses of RT ranging from 45 to 60 Gy have been shown to be inferior to surgery with respect to local control rates [95]. In the pooled analysis of the Cooperative Ewing Sarcoma Study (CESS 81, CESS 86) and the European Intergroup Cooperative Ewing Sarcoma Study (EICESS 92), the incidence of local failure was 26.3% after definitive RT and 7.5% after surgery ± PORT [96]. In a recent study, Laskar et al. evaluated radiation dose escalation in nonmetastatic unresectable extracranial ES using conformal and high-precision RT in a single-institution randomized controlled trial [97]. Patients were randomized to receive standard-dose RT (SDRT; 55.8 Gy/31 fractions) versus escalated-dose RT (EDRT; 70.2 Gy/39 fractions). At a median follow-up of 67 months, the 5-year LC, EFS, and OS for the entire cohort were 62.4%, 41.3%, and 51.9%, respectively. The 5-year LC was significantly better in EDRT compared with SDRT (76.4% vs. 49.4%; *p* = 0.02), but the differences in EFS and OS at 5 years (for EDRT vs. SDRT) did not reach statistical significance (DFS 46.7% vs. 31.8%; *p* = 0.22 and OS 58.8% vs. 45.4%; *p* = 0.08). There was a higher incidence of grade >2 skin toxic effects (acute) in the EDRT arm (10.4% vs. 2.1%; *p* = 0.08). In addition, recent studies suggest patients with larger tumors are at increased risk of local failure [98,99]. Both prospective and retrospective studies have evaluated dose escalation for tumors larger than 8 cm. A retrospective review from the University of Florida reported no local failures in patients with pelvic tumors ≥8 cm treated to ≥59.4 GyE [100], while Talleur et al., in a phase II prospective study, also reported no local failures in tumors ≥8 cm receiving an escalated dose of 64.8 Gy [101]. These studies suggest a role of dose-escalation in selected patients with unresectable ES or larger tumors. There is also a potential role of proton therapy for safe dose escalation in these patients, especially for tumors in critical locations, such as the spine [98].

## 6. Escalation of Radiotherapy Using Hypofractionation and SABR

Radiotherapy may be used to treat oligometastatic disease, with the goal of improving survival. A previous study from Houston showed a better 5-year PFS (31.3% vs. 0%) and 5-year OS (37.3% vs. 0%) in metastatic rhabdomyosarcoma patients who received local therapy to all metastatic sites [102]. Stereotactic ablative body radiotherapy (SABR) has emerged as a promising treatment option to escalate radiation dose for patients with limited metastatic burden and some primary tumors. There is an evolving role of SABR in sarcomas, especially RMS and ES. SABR can offer a therapeutic advantage compared to conventional RT with a higher, ablative dose potentially providing durable local control even for relatively radioresistant histologies. In addition, a shorter fractionation schedule allows minimum interruption in systemic therapy and disruption in quality of life. Pediatric patients with recurrent and metastatic cancers can often have substantial local tumor burden and resulting symptoms. In this setting, local tumor control is important in alleviating symptoms and preventing progression, and can have a significant impact on the quality of life of these patients. In a recent report of 48 pediatric and AYA patients treated with 135 SABR, SABR was well tolerated, with 1- and 2-year LC rates of 93.6% and 89.0%, respectively, and a median OS of 16.9 months [2]. The optimal BED_10_ for treating various pediatric tumors with SABR is not well defined, but significantly improved local control was seen in patients who received a BED_10_ > 48 Gy [2]. In addition, Elledge et al. reported an improvement in median PFS (9.3 months vs. 3.7 months; *p* = 0.03) as well as OS (median not reached vs. 12.7 months; *p* = 0.02) when all known sites of metastatic disease were consolidated with SABR compared with partial consolidation [103]. This is also consistent with data from the EURO-EWING trial indicating improved EFS with local therapy to primary and metastatic sites [104]. The recently completed COG trial AEWS1221 evaluated the use of SABR in the treatment of osseous metastatic sites in Ewing sarcoma patients up to a dose of 40 Gy in five fractions (NCT02306161). Despite these reports, few patients with limited metastatic disease are treated with consolidative RT worldwide [105]. Patients with oligometastatic disease have a better survival rate than patients with widely metastatic disease, suggesting that the total consolidation of all metastatic sites in patients with a limited metastatic burden may be associated with better survival outcomes.

## 7. Role of Modern Radiation Techniques

Recent advancements in radiotherapy technology, such as intensity modulated radiation therapy (IMRT), proton therapy and advanced imaging techniques, have revolutionized risk-stratified approaches. These technologies enable more precise targeting of tumors, which helps reduce CTV margins and decreases normal organ doses [106]. The use of protons can minimize the side effects associated with radiation exposure to eloquent parts of the brain. Proton techniques are helpful in sparing normal tissues nearby, but cannot spare structures involved with tumor. Clinical data support improvements in neurocognitive outcomes with protons versus photon beam therapy, even in younger patients [107,108,109,110]. In addition, using protons for CSI can reduce the dose to anterior organs, including heart, gastrointestinal tract, lungs, kidneys, and thyroid [111], and evaluation of long-term toxicity of proton therapy for medulloblastoma suggests decreased cardiac, pulmonary, and gastrointestinal toxicity compared to photon-based treatments [112,113]. Advancements in radiation techniques have resulted in improvements in the late toxicity profile and can help in risk-stratified treatment planning.

## 8. Conclusions and Future Directions

We hereby discuss the role of risk stratification and personalized treatment options for various pediatric cancer patients, with a goal to improve tumor control and decrease late effects of radiation in long-term survivors. We discuss settings in which radiation can be safely omitted or de-escalated, such as Hodgkin lymphoma, Wilms tumor with lung metastases and WNT pathway Medulloblastoma, and settings that warrant treatment escalation, such as rhabdomyosarcoma >5 cm, poor responders to chemotherapy, unresectable Ewing sarcoma patients and oligometastatic disease settings. We also summarize currently enrolling COG and other cooperative group trials.

## Figures and Tables

**Table 1:** Summary of previous and active COG trials.

Diagnosis	Study	Description	Radiation Details
Medulloblastoma	ACNS1422	A Phase 2 Study of Reduced Therapy for Newly Diagnosed Average-Risk WNT-Driven Medulloblastoma Patients	18 Gy CSI with 36 Gy tumor bed boost
Ependymoma	ACNS0831	Phase III Randomized Trial of Post-Radiation Chemotherapy in Patients with Newly Diagnosed Ependymoma Ages 1 to 21 years	54–59.4 Gy
Intracranial Germ Cell Tumor	ACNS1123	Phase 2 Trial of Response-Based Radiation Therapy for Patients with Localized Central Nervous System Germ Cell Tumors (CNS GCT)	30.6 Gy Whole ventricular irradiation plus 23.4 Gy primary site boost
	AGCT1531	A Phase 3 Study of Active Surveillance for Low Risk and a Randomized Trial of Carboplatin vs. Cisplatin for Standard Risk Pediatric and Adult Patients with Germ Cell Tumors	No radiation
	ACNS2021	A Phase 2 Trial of Chemotherapy followed by Response-Based Whole Ventricular and Spinal Canal Irradiation (WVSCI) for Patients with Localized Non-Germinomatous Central Nervous System Germ Cell Tumor	30.6 Gy ventricular and spinal irradiation plus 23.4 Gy primary site boost
Hodgkin Lymphoma	AHOD2131	A Randomized Phase 3 Interim Response Adapted Trial Comparing Standard Therapy with Immuno-oncology Therapy for Children and Adults with Newly Diagnosed Stage I and II Classic Hodgkin Lymphoma	Involved site RT
	AHOD1331	A Randomized Phase 3 Study of Brentuximab Vedotin (SGN-35, IND #117117) for Newly Diagnosed High-Risk Classical Hodgkin Lymphoma (cHL) in Children and Young Adults	Involved site RT
Nasopharyngeal Carcinoma	ARAR2221	A Phase 2 Study Using Chemoimmunotherapy with Gemcitabine, Cisplatin and Nivolumab in Newly Diagnosed Nasopharyngeal Carcinoma (NPC)	61.2 Gy in 34 daily fractions
Rhabdomyosarcoma	ARST1431	A Randomized Phase 3 Study of Vincristine, Dactinomycin, Cyclophosphamide (VAC) Alternating with Vincristine and Irinotecan (VI) Versus VAC/VI Plus Temsirolimus (TORI, Torisel, NSC# 683864) in Patients with Intermediate Risk (IR) Rhabdomyosarcoma (RMS)	Site-dependent, 6.5 weeks
	ARST2032	A Prospective Phase 3 Study of Patients with Newly Diagnosed Very Low-risk and Low-risk Fusion Negative Rhabdomyosarcoma	Site-dependent, 6.5 weeks
	ARST1321	Pazopanib Neoadjuvant Trial In Non-Rhabdomyosarcoma Soft Tissue Sarcomas (PAZNTIS): A Phase II/III Randomized Trial of Preoperative Chemoradiation or Preoperative Radiation Plus or Minus Pazopanib (NSC# 737754, IND# 118613)	45 Gy preoperative radiotherapy; postoperative boost of 21.6 Gy for R2 resection or 16.2 Gy for R1 resection
Ewing’s Sarcoma	AEWS0331	European Ewing Tumor Working Initiative of National Groups Ewing Tumour Studies 1999 (EURO-E.W.I.N.G.99)	Definitive +/− metastatic site irradiation
	AEWS1031	A Phase III Randomized Trial of Adding Vincristine-Topotecan-Cyclophosphamide to Standard Chemotherapy in Initial Treatment of Non-metastatic Ewing Sarcoma	Definitive +/− metastatic site irradiation
	AEWS1221	Randomized Phase 3 Trial Evaluating the Addition of the IGF-1R Monoclonal Antibody Ganitumab (AMG 479, NSC# 750008, IND# 120449) to Multiagent Chemotherapy for Patients with Newly Diagnosed Metastatic Ewing Sarcoma	Definitive +/− metastatic site irradiation
Wilms Tumor	AREN1921	Treatment of Newly Diagnosed Diffuse Anaplastic Wilms Tumors (DAWT) and Relapsed Favorable Histology Wilms Tumors (FHWT)	Flank and/or metastatic site radiation
	AREN0532	Treatment for Very-Low- and Standard-Risk Favorable Histology Wilms Tumor	Flank (10.8 Gy) or whole abdominal (10.5 Gy) radiation, with a 10.8 or 10.5 Gy boost to gross residual tumor, respectively
	AREN0533	Treatment of Newly Diagnosed Higher-Risk Favorable Histology Wilms Tumors	10.8 Gy flank RT for local stage III tumors, with 10.8 Gy boost for patients with gross residual disease after surgery, whole-lung RT 12 Gy for lung nodules, regardless of pulmonary metastatic lesion response at week 6
	AREN0534	Treatment for Patients with Bilateral, Multicentric, or Bilaterally Predisposed Unilateral Wilms Tumor	Flank radiation if indicated 10.8 Gy (19.8 Gy for ≥16 years old), Whole lung irradiation 12 Gy for patients with lung metastasis (10.5 Gy for <12 months old)
